# Mesenchymal Stromal Cells and Cutaneous Wound Healing: A Comprehensive Review of the Background, Role, and Therapeutic Potential

**DOI:** 10.1155/2018/6901983

**Published:** 2018-05-20

**Authors:** Michael S. Hu, Mimi R. Borrelli, H. Peter Lorenz, Michael T. Longaker, Derrick C. Wan

**Affiliations:** Hagey Laboratory for Pediatric Regenerative Medicine, Division of Plastic and Reconstructive Surgery, Department of Surgery, Stanford University School of Medicine, Stanford, CA, USA

## Abstract

Cutaneous wound repair is a highly coordinated cascade of cellular responses to injury which restores the epidermal integrity and its barrier functions. Even under optimal healing conditions, normal wound repair of adult human skin is imperfect and delayed healing and scarring are frequent occurrences. Dysregulated wound healing is a major concern for global healthcare, and, given the rise in diabetic and aging populations, this medicoeconomic disease burden will continue to rise. Therapies to reliably improve nonhealing wounds and reduce scarring are currently unavailable. Mesenchymal stromal cells (MSCs) have emerged as a powerful technique to improve skin wound healing. Their differentiation potential, ease of harvest, low immunogenicity, and integral role in native wound healing physiology make MSCs an attractive therapeutic remedy. MSCs promote cell migration, angiogenesis, epithelialization, and granulation tissue formation, which result in accelerated wound closure. MSCs encourage a regenerative, rather than fibrotic, wound healing microenvironment. Recent translational research efforts using modern bioengineering approaches have made progress in creating novel techniques for stromal cell delivery into healing wounds. This paper discusses experimental applications of various stromal cells to promote wound healing and discusses the novel methods used to increase MSC delivery and efficacy.

## 1. Introduction

An open wound is a loss of continuity of the epidermis, caused by mechanical, chemical, biological, or thermal injuries. Open wounds can be superficial involving the epidermis and varying degrees of dermis, or full thickness extending to the subcutaneous layer. Cutaneous wound healing is a highly organized physiological process that restores the integrity of the skin following injury. It involves the interplay between various populations of cells and is typically categorized into three overlapping phases: inflammation, proliferation, and maturation [1–3].

The highly coordinated wound repair process is susceptible to interruption or failure by multiple factors which can result in nonhealing wounds. Chronic wounds are defined as those which persist for at least three months and are generally classified as vascular, diabetic, or pressure ulcers. They usually occur due to characteristics of the wound or patient physiology or as a complication of a disease process, all of which prolong or exacerbate the inflammatory process and prevent dermal or epidermal cells responding to regenerative stimuli [4]. Cutaneous injury that penetrates beyond the epidermis in adult human skin is repaired by a highly evolved fibroproliferative response that quickly restores the skin barrier but results in the formation of a scar. Scarred skin lacks dermal appendages, such as sebaceous glands, hair follicles, and sensory nerve receptors [1], and has a reduced tensile strength [5], which alter its visual appearance and impact its normal functions.

Wound healing represents a significant challenge in plastic surgery. Chronic wounds cause substantial patient morbidity, with detrimental effects on patient quality of life, increasing pain, stress, depression, and social isolation [6]. More than six million people suffer with chronic skin wounds every year in the United States alone [7], and with the aging population and increased incidence of diabetes and obesity, this disease burden is increasing [8]. Current standards of wound care focus on identifying and removing precipitating or aggravating factors with the hope of reducing inflammation and allowing the healing cascade to proceed [1, 9]. These treatments are often expensive, time-consuming, and inefficient, and more than 50% of chronic wounds are refractory to conventional treatments [10]. Despite the deleterious consequences of fibrosis and scar tissue formation, there are no effective treatments for scarring [10]. The annual worldwide market for advanced wound care products to reduce scarring and promote healing of long-term wounds is in excess of $20 billion [8].

Given the significant medical and economic burdens, there is a paramount need to develop therapies to overcome the current barriers in wound care. A new therapy for wound healing and regeneration gaining momentum in the past few years is the use of mesenchymal stromal cells (MSCs). MSCs exist in normal skin and play a critical role in wound healing; therefore, application of exogenous MSCs was proposed to promote regenerative healing of wounded skin [11]. This chapter addresses the definition of MSCs, their role in endogenous wound healing, the therapeutic use of MSCs, and the mechanisms by which MSC-based therapies may impact skin healing outcomes.

## 2. Mesenchymal Stromal Cells (MSCs)

**Figure 1:**
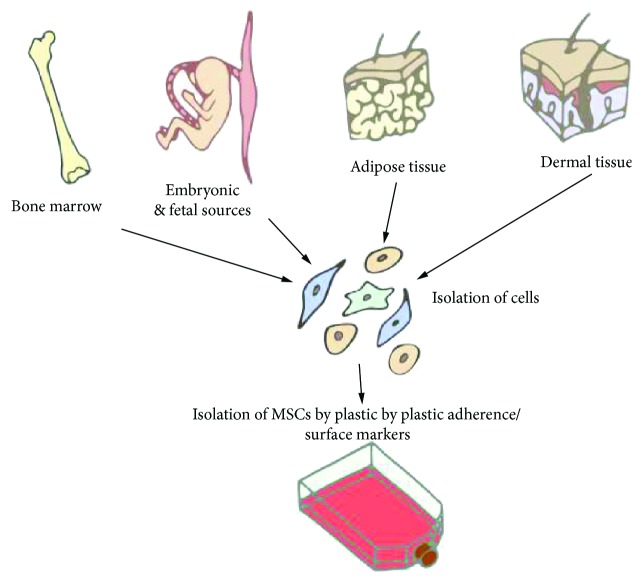
Mesenchymal stromal cells have been isolated from adult tissue including adipose tissue, dermal tissue, and bone marrow, as well as from embryonic and fetal sources. MSCs have been isolated from tissue by their ability to adhere to plastic or by their surface antigen expression (e.g., CD105^+^, CD73^+^, and CD90^+^).

MSCs are progenitor cells of mesodermal origin. MSCs were first isolated from bone marrow in the 1970s [12] by their inherent ability to adhere to tissue culture surfaces like plastic. The cells were notable for their spindle-like shape, the capacity to derive colonies from single cells (“colony forming units-fibroblastic,” CFUs-F), as well as their ability to differentiate into adipocytes, chondrocytes, osteocytes, and fibrous tissue, *in vitro* and *in vivo*. Supporting experimental evidence for these nonhematopoietic multipotent stromal cells was widely reproduced in bone marrow [13], and subsequently, similar cells were described in a range of adult tissues, including adipose tissue [14, 15] and dermal skin tissue [16], as well as from embryonic and fetal sources, such as the amniotic membrane [17], umbilical cord [18–22], and umbilical cord blood/Wharton's jelly [23, 24] (Figure 1).

MSCs have been challenging to precisely define, as the field is complicated by inconsistencies with MSC nomenclature and agreed identifying criteria. MSCs were first termed “osteogenic stem cells” or “bone marrow stromal stem cells” and later labeled “mesenchymal stem cells” [25] and “stromal progenitor cells” [26]. Concerns in the scientific community that these cells are not truly stem cells, given the lack of evidence demonstrating self-renewing capacity *in vivo* [26–28], the International Society for Cellular Therapy (ISCT) in 2005 [27] stated “that fibroblast-like plastic-adherent cells, regardless of the tissue from which they are isolated, be termed multipotent mesenchymal stromal cells, while the term mesenchymal stem cell is only used for cells that meet specified stem cell criteria. The widely recognized acronym, MSC, may be used for both cell populations as is the current practice.” Despite this statement, the term “mesenchymal stem cell” remains widely used and a systematic analysis of bone marrow-derived MSCs (BMSCs) by the European consortium Genostem reported that MSCs are capable of self-renewal, providing evidence that they are stem cells [29]. The stem cell behavior of MSCs remains debated.

The single most characteristic feature of MSCs is their capacity to develop into adipocytes, chondroblasts, and osteoblasts *in vitro.* Demonstrating this trilineage differentiation potential *in vitro* is often performed to confirm MSC identity [30]. However, some reports indicate that MSCs are able to develop into nonmesenchymal lineages, like epidermal and neuronal cells [31–34]. This postulated transgermal potential remains highly controversial; it may be exceedingly rare *in vivo* [35, 36], and cell fusion may account for observed plasticity [37].

There is no specific cell surface marker unique to MSCs, and MSCs are often isolated by “adherence selection.” There is considerable heterogeneity in the expression of cell surface markers between MSC populations, and surfaceome is influenced by rodent strain, MSC isolation and expansion methods [38], and culture conditions [39]. The ISCT attempted to resolve challenges in confirming MSC identity by proposing three minimal criteria for defining human MSCs: (1) the cells must be plastic-adherent when maintained in standard culture conditions using tissue culture flasks; (2) ≥95% of the population must express CD105, CD73, and CD90 and ≤2% must not express CD45, CD34, CD14, CD11b, CD79*α*, or CD19, and HLA class II surface molecules; and (3) the cells must be able to differentiate into osteoblasts, adipocytes, and chondroblasts under standard *in vitro* differentiating conditions [40].

There is a growing body of evidence to suggest that many of the cells exhibiting *in vitro* characteristics of MSCs are identical to or derived from pericytes *in vivo* [41–44]. Pericytes are cells located within the vascular basement membrane of microvessels and capillaries throughout the body, which may indicate there is a common precursor cell type in a wide variety of tissues.

## 3. Endogenous MSCs in Wound Healing

**Figure 2:**
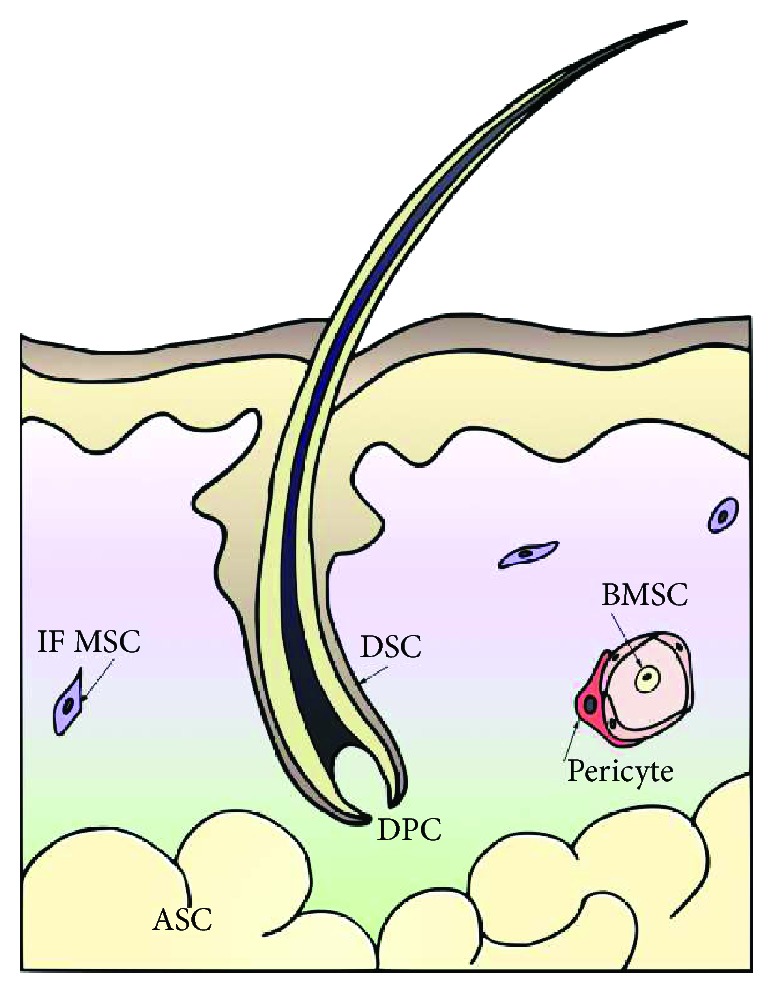
The MSCs involved in normal wound healing. ASC = adipose-derived MSC; BMSC = bone marrow-derived MSC; DPC = dermal papilla cell; DSC = dermal sheath cell; IF MSC = interfollicular MSC.

Endogenous cutaneous MSCs include dermal papilla cells (DPC), at the base of the hair follicle, and the dermal sheath cells (DSC), which surround hair follicle units. DPCs are primarily involved in modulating hair follicle cycling [45, 46], while the DSCs are thought to play a critical role in replacing the dermis in response to injury by differentiating into wound healing fibroblasts [47] (Figure 2). Additional dermal MSCs may be located in the interfollicular dermis [48, 49], and the perivascular pericytes may act as MSCs *in vivo* [50, 51]. Additionally, cutaneous wounding may activate MSCs residing in the adipose tissue. Mature and precursor adipocytes populate the wounded area during the proliferative phase of wound healing, in parallel with fibroblasts. The impaired wound healing of lipoatrophic mice suggests that adipose tissue MSCs have a role in the recruitment of fibroblast and dermal reconstruction. [52] Finally, the BMSCs are also thought to contribute to cutaneous wound healing and are reported to be recruited to wounded tissue in early inflammation and maintained in the reconstructed dermal tissue [53–56].

## 4. Therapeutic Use of MSCs in Wound Healing

MSCs have been exogenously applied to wounds to exploit their physiological therapeutic actions in normal wound healing, and regardless of the caveats in their identity or source, MSCs have been reported to have positive effects on both wound healing and scarring. MSCs have a wide differentiation potential, making them attractive treatment options in regenerative medicine and, over the past decade, have rapidly emerged as treatment of acute and chronic wounds.

Most of the evidence of MSCs in wound healing comes from BMSCs used in animal models, with only a small number of published clinical studies. In an initial case series (*n* = 3), bone marrow aspirate and, in three additional treatments, cultured BMSCs were applied to chronic wounds and were able to decrease wound size and increase dermal vascularity and thickness in histology [57]. In a second case series, autologous BMSCs topically applied to acute surgical wounds and chronic lower-extremity wounds using fibrin spray accelerated healing of acute surgical wounds (*n* = 5) and significantly decreased wound size of chronic venous and diabetic ulcer wounds (*n* = 6) at 20 weeks. Histologically, the MSCs had migrated into the upper layers of the wound bed and differentiated into cells with a fibroblast phenotype. The surface density of MSCs correlated with the reduction in ulcer size [58]. In one of the largest case series, 20 chronic, nonhealing wounds (*n* = 13) were treated with autologous BMSCs impregnated onto a collagen sponge, and ninety percent of the wounds healed completely [59].

A randomized study in 2009 found that cultured autologous BMSCs simultaneously administered topically onto 24 chronic, nonhealing ulcers of the lower extremities and via intramuscular injection into the affected limb significantly decreased wound size (72% versus 25%) and decreased ulcer-associated wound pain at 12 weeks, compared to standard wound care [60]. A second randomized study injected autologous BMSCs intramuscularly into the affected limb and reported improved pain-free walking at 24 weeks and significantly increased ulcer healing rate compared to control treatment [61].

BMSCs are obtained from bone marrow aspiration, which is a safe but painful and invasive procedure, associated with complications such as infection and hemorrhage [18]. Additionally, bone marrow is a limited resource; there is an age-dependent reduction in cell number [62]. The long-term growth and differentiation potential of BMSCs *in vitro* may be limited [63]. Therefore, identification and characterization of alternative sources of human MSCs for wound healing is important. Several different MSCs have been applied to wounds in preclinical investigations of wound healing, including adipose-derived stromal cells (ASCs), dermal MSCs [64], and MSCs from amniotic fluid and umbilical cord. ASCs and dermal MSCs are abundantly available in fat and skin and can be harvested with minimally invasive procedures, and their use lacks ethical controversies making them good alternatives to BMSCs. ASCs and dermal MSCs have similar biological characteristics, immunogenicity, and differentiation potential to BMSCs [15, 16, 65–68], although the clonogenicity and proliferation capacity in long-term cultures of dermal sheath MSCs may exceed that of BMSCs [67]. Additionally, the paracrine expression profiles of all three MSC types vary slightly, and this can differentially affect wound healing. ASCs, for example, may be the preferred MSC population for augmenting angiogenesis [69].

ASCs show promising outcomes in wound healing studies *in vitro* [70, 71] and *in vivo* in animal models [72–76] and are currently being evaluated in clinical trials for their potential to treat burn wounds and ulcers. An initial study has shown that human ASCs, obtained from the debridement of burned artificial dermis, were associated with a high degree of success in healing the wounds of patients who suffered chronic radiation injuries [77]. Likewise, dermal MSCs show beneficial effects on wound healing, both in culture and in animal models [67, 78]. In clinical trials, autologous scalp-end terminal hair follicles grafted into nonhealing leg wounds (*n* = 10) reduced wound size at 18 weeks, with histological evidence of re-epithelialization and vascularization [79]. A randomized controlled trial (RCT) reported that skin grafts containing follicles significantly reduced wound size compared to skin grafts without hair follicles in chronic leg ulcers (*n* = 19) [80].

These promising clinical studies indicate that MSC-based therapies are safe and potentially efficacious, with no indication that any particular MSC tissue origin has an advantage for wound healing over any other [81]. Currently, the clinical trials are few in number and limited by sample size and long-term follow-up. MSCs are thought to exert their therapeutic effects through a multitude of actions, on various cell types, and at all of the phases of the wound healing cascade [81–83]. Current understanding of the mechanisms of MSCs on wound healing and scar minimizing are addressed in the following section.

### 4.1. MSCs in Wound Healing: Inflammation

The first phase of wound healing is the inflammatory phase, which begins at the time of wounding. Activation of the coagulation cascade initiates the release of cytokines and chemokines which stimulate the chemotaxis of neutrophils, followed by macrophages and later lymphocytes, into the wound for debridement. These inflammatory cells, in turn, secrete growth factors and provisional matrix proteins which promote the recruitment of neighboring epidermal and dermal cells to the wound bed [84]. Inflammation controls microbial invasion and clears the wound site of cellular debris; however, prolonged inflammation can result in the formation of scar tissue [85–87]. On the other hand, an absent or inadequate inflammatory response can give rise to chronic, nonhealing wounds [88, 89].

Exogenous BMSCs home to areas of tissue injury. *In vivo* tracing of the fluorescently labeled BMSCs injected into lethally irradiated mice indicates that BMSCs migrate preferentially to areas of cutaneous injury [90]. *In vitro*, human BMSCs show chemotaxis towards inflammatory wound healing cytokines and growth factors, including platelet-derived growth factor (PDGF), insulin-like growth factor-1 (IGF-1), interleukin 1*β* (IL-1*β*), IL-8, interferon-*γ* (IFN-*γ*), stromal cell-derived factor-1 (SDF-1), and tumor necrosis factor *α* (TNF*α*) [91–93]. These migrating BMSCs upregulate chemokine receptors, such as macrophage-derived chemokine (MDC) receptors CCR2, CCR3, and CCR4; tyrosine kinase receptors PDGF receptor and IGF-R; and RANTES (chemokine ligand 5 (CCL5)), which likely coordinate MSC migratory activity [93–95].

Once present at sites of injury, MSCs exert immunosuppressive effects. Wounds treated with MSCs have lower numbers of inflammatory cells and proinflammatory cytokines, such as IL-1 and TNF*α* [96]. Upon exposure to proinflammatory cytokines, including IFN-*γ*, TNF*α*, IL-1*α*, and IL-1*β*, the immunosuppressive phenotype of MSCs becomes activated, and they begin to express chemokines and inducible nitric oxide synthase (iNOS), which suppress T cell responsiveness to inflammation. In response to MSC activity, T cells secrete less IFN-*γ* and more IL-4, and the number of regulatory T cells increases [97–99]. MSCs also regulate the proliferation, differentiation, and function of B cells [100] and natural killer cells [101], causing natural killer cells to secrete less IFN-*γ* [102, 103].

MSCs also suppress the proinflammatory activity of myeloid cells including monocytes [104, 105], macrophages [106–110], and granulocytes [111]. Dendritic cells are modified to secrete less TNF*α* and more IL-10, and their migration, maturation, and antigen presentation activity is lessened [102, 103]. The MSC-conditioned medium is a chemoattractant for macrophages, through the production of macrophage inflammatory protein-1alpha and beta [112], and MSCs skew the phenotype of macrophages toward an anti-inflammatory phenotype M2, characterized by increased phagocytic ability and the upregulation of anti-inflammatory cytokines such as IL-12 and TNF*α* [109, 113, 114]. This macrophage reprogramming is thought to occur through the release of prostaglandin E_2_ that acts on the macrophages through the prostaglandin EP2 and EP4 receptors [106–108]. M2 macrophages promote wound healing by augmentation of fibroblast proliferation and suppress inflammation by inhibiting T cell proliferation [107]. BMSCs and the conditioned medium of BMSCs have also been found to inhibit bacterial growth, in part by secretion of the antimicrobial human cathelicidin hCAP-18/LL-37 protein [115].

### 4.2. MSCs in Wound Healing: Proliferation

The proliferative phase predominates wound healing after two to three days, characterized by the formation of granulation tissue, composed of proliferating keratinocytes and fibroblasts, migrating epidermal cells, and newly synthesized extracellular matrix (ECM), resulting in re-epithelialization and angiogenesis [84, 116–118]. Wounds treated with BMSC and ASC have accelerated re-epithelialization, angiogenesis, and granulation tissue formation [94, 119–125]. Differentiation and paracrine signaling have both been implicated as mechanisms by which BMSCs exert their beneficial effects in the proliferation phase.

Some studies suggest MSCs may differentiate into a variety of skin cells in the healing wounds following transplantation. *In vivo* studies tracing fluorescently labeled BMSCs injected intravenously or topically applied to the wounded skin of mice report the presence of fluorescent cells upon healing that are positive for markers of dermal fibroblasts, endothelial cells, pericytes [126], and epidermal keratinocytes [94, 121, 127]. BrdU-labeled adult MSCs cocultured with heat-shocked sebaceous gland cells, differentiated into sebaceous glands in skin adjacent to the wound [123]. The percentage of BMSC engraftment in the wound, however, is low and decreases with time. Additionally, most studies have concluded evidence of MSC differentiation based on colocalization of green fluorescent protein (GFP) with specific cell phenotype markers; however, colocalization could also occur by MSC fusion to local wound resident cells [128]. Other authors have reported that there is no evidence that MSCs differentiate into phenotypes typical of resident cutaneous cells in the healing skin wound [120].

There is growing evidence that paracrine signaling is the predominant mechanism by which MSCs enhance wound repair. Acellular conditioned medium from BMSCs and ASCs applied to cutaneous wounds of mice accelerated re-epithelialization and wound repair [73, 125, 129]. Proteomic analyses reveal that BMSCs, ASCs, DPCs, DSCs, and umbilical MSCs secrete many known mediators of tissue repair, including growth factors, cytokines, and chemokines [69, 129, 130]. Secreted proangiogenic factors are likely to increase the density of microvessels and cutaneous wound microcirculation and include vascular endothelial growth factor (VEGF), angiopoietin-1, angiogenin, and leptin [71, 76, 96, 125, 131]. Levels of angiogenin and VEGF levels are comparable between MSC populations, but DPCs and DSCs release higher amounts of leptin [69]. The growth factors released include IGF-1, PDGF, epidermal growth factor (EGF), keratinocyte growth factor, basic fibroblast growth factor (bFGF), SDF-1, erythropoietin, transforming growth factor-*β* (TGF-*β*), and hepatocyte growth factor (HGF) [69, 71, 121, 129, 130]. These growth factors promote migration and proliferation of endothelial cells, epidermal keratinocytes, and dermal fibroblasts *in vitro* and significantly influence wound re-epithelialization *in vivo* [71, 73, 125, 129, 132, 133]. ASCs and amniotic fluid-conditioned media facilitate the production of ECM components such as collagen I by dermal fibroblasts, and this is thought to be mediated via the TGF-*β*/SMAD2 pathway [71, 125, 130, 134–136]. MSCs thus have a potent secretome capable of influencing the activation, migration, and proliferation of different cells involved in the wound healing process to promote angiogenesis, epithelialization, and fibroproliferation.

### 4.3. MSCs in Wound Healing: Remodeling and Maturation

Upon wound closure, the injured site undergoes remodeling and maturation phases. This final phase of wound healing can last up to two years depending on wound severity. The ECM synthesized during the proliferative phase is laid down in a disorganized manner. During remodeling, ECM molecules are realigned and cross-linked by fibroblasts. Fibroblasts also replace collagen III with collagen I. The wounds gradually contract as fibroblasts, stimulated by TGF-*β*1 or *β*2 and PDGF, assume a contractile myofibroblast phenotype and deposit smooth muscle actin [137, 138]. Cells and blood vessels that are no longer required are removed via matrix metalloproteinase- (MMP-) mediated remodeling. Eventually, remodeling leads to the formation of an acellular scar [2, 139].

MSC treatment increases wound tensile strength [119], reduces scarring [140], reduces wound contraction [124], and increases collagen expression [141]. Prolonged inflammation can induce fibrosis, and the anti-inflammatory action of MSCs may be responsible for their antifibrotic effects. The relative increase in the ratio of anti-inflammatory M2 to proinflammatory M1 macrophages may help to reduce scarring, as increased M1 macrophage is thought to be a major regulator of impaired wound healing and tissue fibrosis [142]. Reduced scarring may also be a result of accelerated wound closure, improved angiogenesis, and modified collagen deposition. The paracrine signaling of MSCs may also promote an antiscarring environment. MSCs secrete high levels of VEGF and HGF and maintain a higher ratio of TGF-*β*3 to TGF-*β*1. bFGF and HGF enhance the regeneration of dermis in acute incisional wounds [143]. Increased VEGF is associated with scarless repair [144], as is neutralization of TGF-*β*1 and TGF-*β*2 or addition of TGF-*β*3 [145]. Hypoxic conditioned medium of placenta-derived MSC protects against scar formation through increased production of IL-10 and through the inhibition of the proliferation and migration of fibroblasts [146]. Additionally, BMSCs appear to be responsible for the secretion of collagen type III [56], and a higher collagen type III : type I ratio is associated with the scarless wounds characteristic of healing in fetal tissue [147].

## 5. Optimizing MSC Treatment

Optimizing MSC engraftment in the cutaneous wounds is critical to achieving maximal clinical benefit. There is considerable heterogeneity in the delivery protocols, wound models, and MSC populations between published studies making it difficult to determine the impact of timing of delivery, number of cells delivered, and site of delivery on MSC engraftment. MSCs are effective in clinical and preclinical studies when applied immediately after cutaneous wounding [94, 120–123], within 24 hours of injury [119], and when applied to chronic wounds [58, 60, 77, 79, 148]. MSCs have been successfully administered systemically via intravenous injection [94, 119] and locally via direct intradermal injection [121], or through the use of phosphate-buffered saline (PBS) [120], matrigel [121], fibrin polymer [58], or hydrogel seeding [126]. Additionally, MSC-conditioned medium is effective alone [73, 125, 129] and has the advantage of circumventing challenges with MSC engraftment.

Direct injection or topical administration of MSCs through gel matrices can be detrimental for cell survival. Impregnating scaffolding materials with MSCs may provide a microenvironment more suitable for cell adhesion, proliferation, and differentiation [149]. Scaffolds can be made of natural biomaterials, like collagen and hyaluronic acid, the major constituents of the ECM, as well as fibrin, a protein essential for coagulation. These biomaterial scaffolds have high biocompatibility [3] and, in preclinical studies, enhance wound epithelialization and granulation and downregulate inflammation [150, 151]. MSCs used in combination with collagen-based dermal substitutes promote the migration of cells to wounded skin and vascularization of the scaffold *in vivo* in mice [152]. BMSCs and umbilical cord MSCs cocultured with HaCT, a keratinocyte cell line, distributed on collagen scaffolds, distributed more homogenously within the wound, and expressed higher numbers of ECM proteins and growth factors compared to MSCs applied on scaffolds without collagen, indicating that collagen-based scaffolds may direct cell proliferation and ECM remodeling [153]. MSCs delivered in collagen- and fibrin-based biomedical devices show promise in nonhealing and chronic wounds in clinical studies [154]. Collagen sponges impregnated with BMSCs (*n* = 20 patients) completely healed chronic burns, lower-extremity ulcers, and decubitus ulcers [59]. BMSC delivered on a fibrin glue and collagen matrix also completely or significantly closed diabetic ulcers [155]. Scaffolds can also be made of polysaccharides like chitosan, which have antimicrobial and homeostatic activity, and are able to stimulate the proliferation of fibroblasts, tissue granulation, re-epithelialization, and collagen deposition [3]. Synthetic polymers, such as those made of polyethylene glycol, are also biocompatible and biodegradable, and their properties, such as strength and degradation rate, can easily be manipulated [156]. Growth factors and therapeutic agents can be used with the scaffolds. MSCs in porcine skin substitute decreased wound size, and FGF could be delivered to further accelerate wound healing [157]. Porous collagen scaffolds loaded with the chemotactic cue stromal cell-derived factor-1*α in vivo* promoted the recruitment of MSCs to the injured area [158]. Additionally, applying MSCs with other skin cells may reflect the multicellular composition of skin and promote an environment more conducive to healing. Coculturing human epidermal skin cells and DPCs on a porcine acellular matrix produced a more structured multilayered stratified epidermis when compared with the culture of either of these cells or dermal fibroblasts alone [159].

Recent technological advancements have led to the creation of micro- or nanostructured scaffolds by mechanisms such as electrospinning and freeze drying. These scaffolds can provide mechanical support and protection to the injured areas, with physical characteristics, such as size, network organization, and mechanical properties, that precisely mimic native skin. Electrospun nanofibers of collagen and poly(lactic-co-glycolic acid) (PLGA) seeded with BMSCs promoted collagen synthesis and re-epithelialization in full-thickness skin wounds in a rat model [160]. Bilayer nanofibrous polymer fibers of poly(*ε*-caprolactone-co-lactide)/poloxamer (PLCL/poloxamer) combined with dextran and gelatin substrates were found to more accurately mimic the multilayer structure of the skin [161]. Nanofibers of polyvinyl alcohol (PVA), gelatin, and azide were able to promote the differentiation of ASCs to keratinocytes [162]. BMSCs cultured on electrospun nanofibers of collagen and poly(l-lactic acid-co-e-caprolactone) (PLLCL) had an increased proliferation rate, and their fibroblastic morphology gradually progressed toward that one of epidermal cells [163]. Chitosan-electrospun mats with cellulose or chitin nanocrystals are biocompatible and noncytotoxic scaffolds and may promote ASC proliferation [164, 165]. Polymer structures made of poly(3-hydroxybutyrate-co-hydroxyvalerate) (PHBV) and seeded with ASCs have been shown to withstand forces of contraction *in vivo* and enhance the granulation, re-epithelialization, and vascularization of wounded skin, resulting in skin with a well-organized dermal matrix with sebaceous glands and hair follicles and less scarring after 28 days [166].

Computational modeling of cell behavior and the biophysical processes within the healing tissue can inform the design of efficient scaffolds sensitive to these complex processes, to promote a regenerative wound healing environment. Processes such as the changes in fluid composition, mechanical stress, cell density, and nutrient levels, for example, can be modeled, to create scaffolds timely release of molecules, efficient cell spreading, transport and consumption of nutrients, and controlled scaffold degradation [149]. Despite advances in the development of biomedical dressings and current insights into skin healing, more comprehensive models specifically conceived for skin regeneration are yet to be devised [149].

Additionally, MSCs can be differentiated or preconditioned for therapeutic potential. The differentiation of MSCs towards specific cell fates can be enhanced through the introduction of transcription factors. MSCs have successfully been differentiated into neural, pancreatic, and endothelial cells [167]. A wound environment more permissive for differentiation can also be created to maximize the performance of stem cell-based approaches. Wounded tissue is hypoxic due to the disruption of the blood vessels and increased oxygen consumption by the local cells [168]. Chronic wounds may be especially subject to metabolic perturbations including ischemia or hyperglycemia, which may influence MSC behavior. Hypoxic preconditioning of cultured MSCs has been found to beneficially influence MSC activity, with no effect on MSC viability [169], except if prolonged [170]. The conditioned media from ASCs cultured under hypoxic conditions, maintained in normoxic environment, increases MSC migration through matrigel [171] and on tissue culture plastic [172] by upregulating MMPs. Hypoxic culturing improves MSC proliferation, clonogenicity, survival, and engraftment [95] and accelerates wound closure [73]. Conditioned media from ASCs harvested under hypoxia promotes collagen synthesis compared with that harvested under normoxia [73]. Low oxygen-level conditions increase hypoxia-inducible factor- (HIF-) 1a synthesis, which activates a number of genes involved in angiogenesis and wound healing, including PDGF, TGF-*β*1 and TGF-*β*3, and SDF-1 [173–177]. The growth factors VEGF and bFGF may be potential mediators of this increased efficacy of hypoxic conditioned media of BMSCs, ASCs, and amniotic fluid-derived MSCs [73, 178, 179]. VEGF and bFGF are proangiogenic and induce proliferation and migration of keratinocytes, human dermal fibroblasts, endothelial cells, and monocytes. Hypoxic culture results in strong secretion of immunomodulatory molecules such as programmed death ligand-1 and indoleamine 2,3-dioxygenase and modulates inflammatory cell recruitment, which positively impacts the inflammatory phase of wound healing, favoring reduced inflammation and regenerative wound healing [180].

## 6. Current Limitations and Future Directions

Despite advancements in MSC-based therapy, there are a number of challenges to overcome before MSCs can be used for effective wound healing. The heterogeneity in MSC surface receptor expression and MSC behavior within and between studies emphasizes the importance of consistent criteria to define MSCs, standardized protocols for isolation and expansion, and standard *in vivo* assays to demonstrate their identity prior to therapeutic administration. Most of the mechanisms discussed have been studied in rodents, but animal physiology cannot always be extrapolated to humans. The therapeutic potential of MSCs in human wounds is currently only supported by a small number of clinical studies, and, while results are promising, they are limited by small sample sizes, short follow-up periods, and lack of randomized controlled trials. There are several trials currently recruiting patients to examine the longer-term outcomes of BMSC therapy on diabetic and venous ulcers. Current understanding of MSC mechanisms is still unfolding, and further investigation into how MSC-derived signals target cells and cellular responses spatiotemporally *in vivo* is needed.

## 7. Conclusion

MSC-based therapy is emerging as a promising technique able to promote wound healing and minimize scarring. Their easy and convenient isolation, extensive proliferation potential and differentiation capacity, and lack of significant immunogenicity and ethical controversy make MSCs attractive therapeutic agents. MSCs promote healing in all phases of wound repair. They migrate to sites of cutaneous injury and, primarily through paracrine signaling, suppress inflammation and stimulate the proliferation and differentiation of resident progenitor cells including fibroblasts, endothelial cells, and epidermal cells. MSCs modify their activities and functions depending on the biomolecular context and exposure to biochemical factors characteristic of an injury environment such as hypoxia. Current challenges with the use of MSCs concern the lack of universally accepted criteria for defining the MSC phenotype or their functional properties, and further clinical trials are needed to demonstrate the therapeutic potential of MSCs in a larger number of patients.
